# Meta-analytical biomarker search of EST expression data reveals three differentially expressed candidates

**DOI:** 10.1186/1471-2164-13-S7-S12

**Published:** 2012-12-07

**Authors:** Timothy H Wu, Lichieh J Chu, Jian-Chiao Wang, Ting-Wen Chen, Yin-Jing Tien, Wen-Chang Lin, Wailap V Ng

**Affiliations:** 1 Institute of Biomedical Informatics, National Yang Ming University, Taipei, Taiwan, R.O.C; 2 Molecular Medicine Research Center, Chang Gung University, Taoyuan, Taiwan, R.O.C; 3 Department of Biotechnology and Laboratory Science in Medicine and Institute of Biotechnology in Medicine, National Yang Ming University, Taipei, Taiwan, R.O.C; 4 Bioinformatics Center, Chang Gung University, Taoyuan, Taiwan, R.O.C; 5 Institute of Statistical Science, Academia Sinica, Taipei, 11529, Taiwan, R.O.C; 6 Institute of Biomedical Sciences, Academia Sinica, Taipei, Taiwan, R.O.C; 7 Center for Systems and Synthetic Biology, National Yang Ming University, Taipei, Taiwan, R.O.C

## Abstract

**Background:**

Researches have been conducted for the identification of differentially expressed genes (DEGs) by generating and mining of cDNA expressed sequence tags (ESTs) for more than a decade. Although the availability of public databases make possible the comprehensive mining of DEGs among the ESTs from multiple tissue types, existing studies usually employed statistics suitable only for two categories. Multi-class test has been developed to enable the finding of tissue specific genes, but subsequent search for cancer genes involves separate two-category test only on the ESTs of the tissue of interest. This constricts the amount of data used. On the other hand, simple pooling of cancer and normal genes from multiple tissue types runs the risk of Simpson's paradox. Here we presented a different approach which searched for multi-cancer DEG candidates by analyzing all pertinent ESTs in all categories and narrowing down the cancer biomarker candidates via integrative analysis with microarray data and selection of secretory and membrane protein genes as well as incorporation of network analysis. Finally, the differential expression patterns of three selected cancer biomarker candidates were confirmed by real-time qPCR analysis.

**Results:**

Seven hundred and twenty three primary DEG candidates (p-value < 0.05 and lower bound of confidence interval of odds ratio ≧ 1.65) were selected from a curated EST database with the application of Cochran-Mantel-Haenszel statistic (CMH). GeneGO analysis results indicated this set as neoplasm enriched. Cross-examination with microarray data further narrowed the list down to 235 genes, among which 96 had membrane or secretory annotations. After examined the candidates in protein interaction network, public tissue expression databases, and literatures, we selected three genes for further evaluation by real-time qPCR with eight major normal and cancer tissues. The higher-than-normal tissue expression of COL3A1, DLG3, and RNF43 in some of the cancer tissues is in agreement with our *in silico *predictions.

**Conclusions:**

Searching digitized transcriptome using CMH enabled us to identify multi-cancer differentially expressed gene candidates. Our methodology demonstrated simultaneously analysis for cancer biomarkers of multiple tissue types with the EST data. With the revived interest in digitizing the transcriptomes by NGS, cancer biomarkers could be more precisely detected from the ESTs. The three candidates identified in this study, COL3A1, DLG3, and RNF43, are valuable targets for further evaluation with a larger sample size of normal and cancer tissue or serum samples.

## Background

One of the key aspects in the study of cancer is to understand the principles and mechanisms of gene expression variation contributing to cancer genesis and progression. The identification of genes differentially expressed between normal and cancer cells/tissues is not only helpful for designing diagnostic and therapeutic procedures, but also for understanding cancer biology as a whole. In this regard, DNA microarrays have been the dominating platform in the high-throughput study of cancer transcriptomes since their emergence in the mid-1990s [1,2]. However, there are several drawbacks, which include: high background level signals resulting from cross-hybridization [3,4]; difference in hybridization properties due to different probe sequences; limited dynamic range due to background level and saturation, and difficulty in detecting splicing isoforms and unknown genes. For these reasons, with the advancement of the next generation sequencers, we are seeing high-throughput transcriptome mapping and quantifying method, also known as RNA-Seq, to begin to supersede microarray in expression profiling. However, RNA-Seq experiments are relatively demanding in terms of time, cost, and computation equipment. Experimental differences between different sequencing platforms may complicate transcriptome analysis with multiple tissue sources. Since exploring meta-analysis from traditional digital expression data such as EST derived from cDNAs [5-8] is more feasible, this study may serve as a precursor to more complicated experiments.

Originally primarily aimed for cataloging of transcript repertoire, ESTs from large-scale cDNA sequencing projects such as Cancer Genome Anatomy Project (CGAP), Human Cancer Genome Project (HCGP), and Cancer Genome Project (CGP) also allow searching for differentially expressed genes (DEGs) in specific tissue types or in whole genomes [9-11]. Several *in silico *analysis tools such as NCBI Unigene cDNA xProfiler [12], CGAP Digital Differential Display (DDD) [13], and CGAP Digital Gene Expression Displayer (DGED) [14] are available online allowing the analysis of publicly available data. While standard statistical methods such as Fisher's exact test for finding DEGs in two-class problems (e.g. cancer vs. normal) or Pearson's correlation are commonly used [9], there are also specially developed methods for finding DEGs in the landscape of digital signals for two-library problems [15,16] or for multiple libraries [17]. The online tools as well as the statistical methods remain useful to this day in EST or even RNA-Seq projects [18-23]. Aside from searching for DEGs, the searches for gene transcript isoforms specific to particular libraries were also demonstrated and many of these attribute differentially expressed isoforms to human cancers [24-31].

In spite of the successful applications, these tools or methods are not without limitations. xProfiler reports differential expression in an all-or-none manner where only a list, but not statistical quantification, of candidates is reported. DDD allows quantification using Fisher's exact test. However, the nature of the test dictates that comparisons of three or more libraries involve multiple pair-wise comparisons, and thus there are no easy comparisons of library specific genes. DGED uses a Bayesian approach to find DEGs, but it is also pair-wise. The reported "odds ratio" is perhaps better described as "relative risk" and may be biased with unequal sampling. Another popular and useful Bayesian-based method originally developed for EST analysis by Audic and Cleverie [15] is also popular for RNA-Seq data. It is less conservative than Fisher's exact test, but it also does not apply to multi-class problems. The multi-class comparison method established by Stekel *et al*. [17] finds specificity in one condition out of all and is useful in application such as finding DEGs in multi-tissue libraries. However, in the search for cancer DEGs, a subsequent analysis of differential expression between cancer and normal libraries of the tissue of interest may not yield fruitful results due to the possible scarcity of EST sampling in the particular tissue type. On the other hand, the naïve method of pooling all data into the two-class problem of normal versus cancer when searching for differentially expressed genes or differentially splice variants [27] risks introducing bias. In extreme cases, one may encounter the fallacy of Simpson's paradox [32] where genes in reality more active in the normal condition appear to be more so in the cancer condition (discussed later in this paper).

We now report on the application of a computational and integrative approach to analyze cancer differentially expressed genes (DEGs). The statistical method we employed is Cochran-Mantel-Haenszel statistics (CMH) [33] and to the best of our knowledge has not been applied in this context. Instead of pooling all normal and all cancer ESTs from different tissue types to fit into a two-class problem as by using the 2 by 2 contingency χ^2 ^test or the Fisher's exact test, CMH allows original stratification of libraries in their respective tissue types, yet exhaustively analyzes expression between cancer and normal conditions across all tissue types. The method is an extension to χ^2 ^test which, in our application, measures the association between cancer and gene expressions, adjusting for the tissue confounding factor. This approach allows one to find genes that are overall differentially expressed in cancer, or multiple-cancer genes, irrespective to a specific tissue type. The method is demonstrated in this paper to exhaustively analyze ESTs from the dbEST database [34]. To the best knowledge of the authors, such an all-inclusive, whole-transcriptome analysis has not been redone in recent years now that more ESTs than ever are available.

Our filtering of EST libraries was also more rigorous than many previous studies. Notably, we excluded the ORESTES (open reading-frame EST sequencing) libraries [35] on which a normalization procedure had been applied. Libraries from cell line were also excluded owning to their unrepresentativeness of primary cancer cell transcriptomes. Our analysis pipeline further focused on enrichment of the DEGs by cross examination with expression data of a different platform, *i.e*. the microarray data, and selecting for membrane and secretory associated protein genes since we intend to find therapeutic targets or biomarkers, and conducting STRING (The Search Tool for the Retrieval of Interacting Genes) network analysis to show the cancer enriched clusters [36]. With real-time qPCR validation, we have identified three candidates that are inclined to express in cancer across more than one tissue types. We hope such a meta-analytical and multiple-tissue comparison can serve as an exploratory experiment for future multi-library or multi-tissue study of other digital sources such as RNA-Seq.

## Methods

### Overview

**Figure 1:**
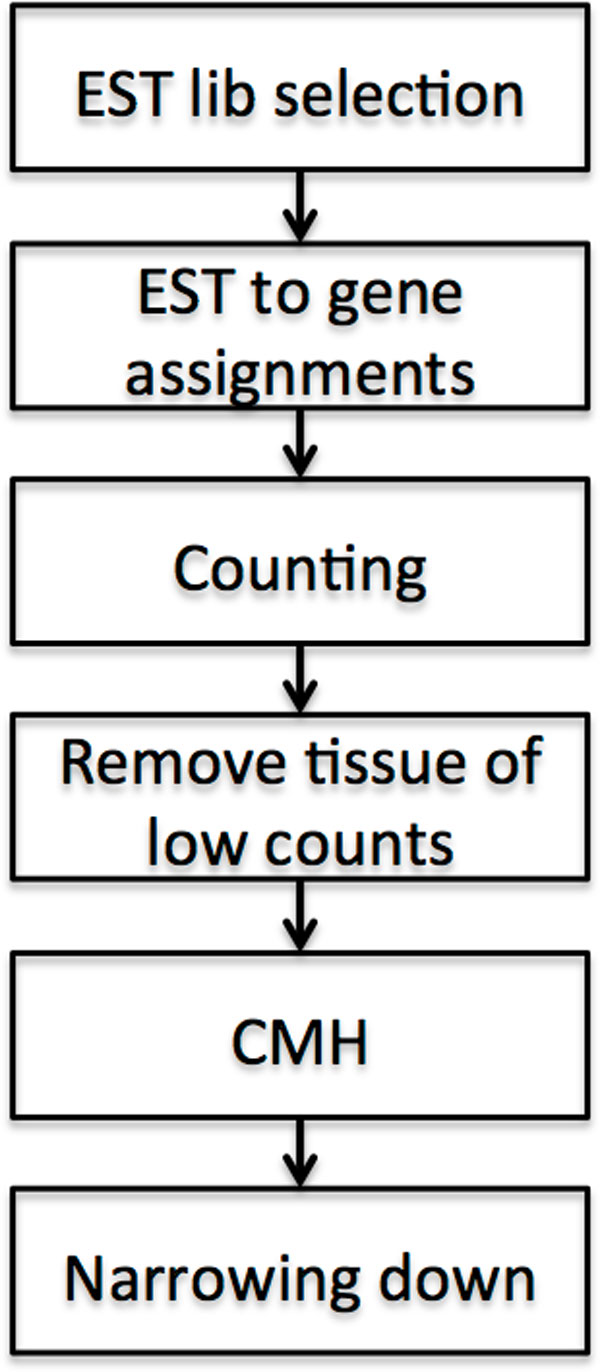
**The basic steps in searching for differential expression genes**. EST library selection involves selection of suitable EST clone libraries, EST to gene assignment, counting the results, remove tissue categories with low counts, statistical analysis with CMH and the narrow-down of differentially expressed genes (DEGs). The narrow-down procedures includes cross referencing with public microarray data, annotating membrane and secretory proteins, analyzing with String network, and for a few selected genes, validate the expression in different tissues by RT-qPCR.

Our approach was to exploit the entire collection of human EST sequences from dbEST [34] to obtain transcripts from different type of cells/tissues/organs. The assumption was that the activities of the genes can be represented by their transcripts, and also reflected by the number of representing ESTs in the NCBI dbEST database, given that a large number of mRNAs (cDNAs) were sequenced. Pertinent sequences from different sources were matched to genes and tallied together. Through the annotation of each EST record, we obtained the tissue type and condition type (normal or cancer) from which it was derived. With the information, we then had the entire gene transcription profile for all the tissues and conditions. Next, cross examining data of other sources including microarray data, secretory and membrane associations as well as analyzing protein associations with STRING [36] allowed us to narrow down the list of candidate genes. The process is illustrated in Figure 1.

### Human gene reference sequence preparation

The NCBI Reference Sequences (RefSeq Release 38, November 11, 2009) [37] were downloaded from its ftp site [38]. *Homo sapiens *RefSeq records were selected and subjected to repeat masking via RepeatMasker [39].

### Human EST sequence preparation and library filtering

Human EST data (Released on December 11, 2009) and their cDNA library information were downloaded from NCBI dbEST database [34] and CGAP [40]. Program in Python language was written to mark for discard the unsuitable libraries when the keywords such as "enrichment", "subtract", "pcr", and "normalized" were found in the DESCR, UNIQUE_PROTOCOL, or KEYWORDS fields of the library information. An arbitrary cutoff of > 400 was chosen to the highly unrepresentative libraries (approximately 7,000 libraries constituting approximately 650,000 ESTs were discarded as a consequence). To curb from incorrect inclusions or exclusions, we finalized the process with manual curation. Libraries made from mixed tissues or cell lines were also discarded. The final libraries from CGAP were manually classified into 48 different tissue types and two different conditions, normal and cancer.

### EST to gene assignment

The BLAT alignment tool was used to align ESTs to RefSeqs as a mean to assign ESTs to genes [41]. The criteria of having an identity of 95% or above and the minimum length of 100 nucleotides were set for a match. The RefSeq match with the highest identity was assigned for the EST. If two RefSeq matches shared exactly the same identity, the program chose the first encountered.

### EST count and summarization

The procedure attributes each transcript represented by RefSeq its expression profile across different tissue and condition types based on EST assignment counts. Each EST has its corresponding tissue type and condition type classification, based on its source clone library. For example, a transcript with an aligned EST from a lung cancer clone library is one expression count each in tissue type lung and condition type cancer. This way, after all ESTs were counted, each transcript has a profile of expression across various libraries and conditions. Expressions from different transcript variants of the same gene were pooled to obtain a single gene expression. The raw counts were thus made into transcription profile for each gene for further statistical analysis.

### Statistical evaluation of cancer candidates

Cochran-Mantel-Haenszel statistics (CMH) was applied to evaluate cancer differential expression of each gene. To evaluate each gene, other genes were pooled as "other genes" to create a 2 × 2 × *k *table consisting of data from tissue-condition cross, where *k *was the number of tissues × 2 (two conditions). A contrived example of 2 × 2 × *k *table where *k *is 2 is shown in Table 1. Gene A is the gene under study while other genes are pooled together as "other genes". Only Tissue I and Tissue II columns are calculated in CMH. The pooled ones are not part of the analysis. Akin to Fisher's exact test, the test assumes that "other genes" should consist mostly of genes not differentially expressed between normal or cancer conditions. Or, some of them are DEGs for one condition, but they are at least partly canceled out by DEGs for the other. In any case, the imbalances of cancer counts to normal counts in the second row is regarded as owning to sample bias and it serves as a metric against which Gene A is measured. By continuously isolate values for gene currently under study while pooling all other genes to the second row, an odds ratio and a confidence interval is calculated for each gene. Genes with a p-value < 0.05 and an lower bound of confidence interval of odds ratio ≧ 1.65 are selected for further analyses.

**Table 1:** A hypothetical EST count table demonstrating CMH analysis and also a contrived example of Simpson's paradox.

	Tissue I		Tissue II		Pooled	
			
	Normal	Cancer	Normal	Cancer	Normal	Cancer
Gene A	280	580	20	20	**300**	**600**
Other genes	**20,000**	**80,000**	**380,000**	**620,000**	400,000	700,000

This hypothetical case serves both as an example of how Cochran-Mantel-Haenszel (CMH) is applied as well as the occurrence of Simpson's paradox. Gene A is the gene under investigation. Expressions from all other genes are pooled into the "other genes" row. Bold typeface indicates columns showing higher cancer vs. normal propensities. CMH is applied on the stratified tissue columns (but not on the pooled data). A casual observation involving only the pooled data would suggest Gene A as having higher expression in cancer (*X*^2 ^test p-value close to 0 when analyzing only the pooled). However, a closer inspection on each of the tissue columns reveals otherwise. The observed difference between cancer and normal of the "other genes" is theoretically mostly due to sampling bias.

### Microarray cross reference

Human U133 Plus 2.0 GeneChip array CEL data were downloaded from Gene Expression Omnibus (GEO) [42]. When computing power allows, the data were processed with AffyPLM [43] using its three-step procedure of processing background signals with GCRMA, normalizing signals with quantile normalization, and summarize probe signals with medium polish. For large experimental datasets that were computationally infeasible for us, we used justRMA from the Affy package [44]. For experimental dataset without raw CEL data, we obtained the pre-processed matrix files via GEOQuery [45]. Regardless of the source of array signal processing, we analyzed the genes for differential expression with Limma [46]. Differentially expressed gene candidates with p-value < 0.05 and logFC > 1.0 were selected and crossed with genes from EST profiling with statistical evaluation. For each array, the significant genes were crossed with our EST profiling results. The union of these intersecting genes was selected for further evaluation.

### Annotation of secretory proteins

To identify our differentially expressed genes with secretory annotation, a list of 3,975 proteins with secretory annotation originated from the conglomeration of data from Uniprot (1,632 unique proteins) [47], Human Plasma Proteome Organization (HUPO) (889 proteins), and Secreted Protein Database (SPD) (4,142 proteins) [48]. This list was matched against DEGs to give them secretory annotation.

### Annotation of membrane proteins

Membrane protein annotations were gathered from five sources - TOPDB (283 proteins) [49], LOCATE (2629 proteins) [50], PDB_TM (41 proteins) [51,52], OPM (107 proteins) [53], and MPDB (23 proteins) [54] - to generate a unique list of 2,767 membrane proteins. Any DEGs on this list would confer it a membrane annotation.

### Validation of tissue expression profiles of candidate genes

TissueScan™ Cancer Survey Panel 96-I qPCR array panel (Origene Technologies, Rockville, MD) containing the cDNAs of 3 normal and 9 cancer tissues each from 8 organs (breast, colon, kidney, liver, lung, ovarian, prostate, and thyroid) was used to examine the expression profiles of selected cancer differentially expressed gene candidates. Real-time qPCR analyses with the Taqman^® ^Gene Expression Assay kits (Applied Biosystems, Foster City, CA) and FAM- and VIC-labeled target genes and HPRT1 internal control primers, respectively, were performed according to the manufacturer's suggested procedure on an Applied Biosystems Prism 7500 system. Relative specific gene expression was quantified by normalization against the HPRT1 with the ΔCT method. Gene expression changes were quantified as 2 ^- (CT gene - CT control)^.

## Results

### Human ESTs selection and tissue distribution

The basic steps of our analysis are illustrated in Figure 1. A total of 8,296,089 human EST sequences (Dec. 11, 2009 release) were downloaded from the NCBI. Despite the size of the data, not all ESTs are relevant for our gene expression analysis. After screening the 8,907 EST libraries as described in the methods section above, 8,447 unsuitable libraries, the preparation of which involved PCR amplification, normalization, subtraction, etc. or originated from cell lines, were discarded. The remaining 460 libraries consisted of 2,386,536 EST sequences representing approximately a third of all the downloaded human ESTs.

After BLAT alignment of the 2,386,536 ESTs to 44,513 gene transcripts from RefSeqs, approximately 1,644,960 (68.92%) ESTs with at least 100 nucleotides matched to RefSeqs were detected. An examination of the sources of the matched ESTs indicated that the representativeness of each tissue is skewed and that the brain is the most represented out of all tissues. Among the 48 different tissues, brain ESTs constituted 26% of all matched ESTs, uterus (6.40%) ranked second, followed by testis (5.91%), placenta (4.33%), pancreas (3.99%), muscle (3.88%), liver (3.51%), kidney (3.52) and others each below 3% (see Additional file 1). Similarly, condition type (normal and cancer) representation was also skewed. Normal tissue type had 1,251,883 ESTs combined, and cancer tissue had 393,077 ESTs in the ratio of roughly 3 to 1. Originally before filtering out those from the cell lines, there were more cancer ESTs and the ratio of normal ESTs to cancer ESTs was roughly 1 to 3. This showed how much more rigorous our filtering was. Unfortunately, this also meant we had a much smaller dataset to work with.

The unequal distribution of the 1,644,960 matched ESTs in different tissue types caused some tissue types to be ill-represented. For example, the number of brain EST hits dominated over other tissue types. On the other hand, spinal cord had the least count with 430 EST hits. The latter had little value for our application. Therefore, we only took a tissue type into consideration when its total EST hit count was above the cut-off of 20,000. Considering that the human genome has approximately 22,000 genes, the cut-off still did not allow "deep" probe into gene expression. Nevertheless, the method we employed did not attempt to identify specific gene expression in one particular tissue; therefore, the problem was mitigated.

We also categorized ESTs according to their clone library classification, to either be from normal or from cancer. Sometimes a certain tissue-condition type was so under-represented that the information was not trustworthy. For example, adipose had 10,362 normal hits but only 440 cancer hits, and heart tissue had 22,179 normal hits but no cancer hits. For these cases, data was kept throughout the analysis. But these data did not make contribution to our analysis.

Since our EST assignments were made to transcripts represented by RefSeq sequences, when the entire assignment procedure was done, each transcript variant had its expression profile across all tissue-condition types. Due to the lack of enough ESTs data, differentiating between different splicing variants of the same gene was not feasible. We had to pool expression from different splicing variants into a single expression profile representing the gene.

### Analysis of differentially expressed genes

Due to the small sample size (EST counts), it was only realistic to evaluate gene expression based on all ESTs of all tissues. However, tissue type was a confounder. If all counts for each gene were pooled as "normal" or "cancer" regardless of the tissue of origin, the count would be incorrect. To solve both the sample size and the tissue confounder problems, Cochran-Mantel-Haenszel statistical method was employed to identify genes with differential expression as described in the method. We used the arbitrary cut-offs of p-value < 0.05 and odds ratio ≧ 1.65 to obtain a primary set of candidates. As a result, a total of 723 cancer differentially expressed gene candidates were selected. The 1.65 cut-off is chosen based on a good coverage to a list of well-known biomarkers or genes known to associate with cancer (Table 2).

**Table 2:** EST counts and odd ratios of 11 well-known cancer-related genes present in our list of DEGs.

Gene symbol	Description	Total	Normal	Cancer	Odds ratio
BCAN	Homo sapiens brevican	391	79	312	10.4
KRT14	Homo sapiens keratin 14	205	40	165	9.1
KRT16	Homo sapiens keratin 16	41	7	34	7.8
MMP11	Homo sapiens matrix metallopeptidase 11 (stromelysin 3)	68	20	48	5.3
MUC1	Homo sapiens mucin 1, cell surface associated	69	30	39	4.2
VEGFA	Homo sapiens vascular endothelial growth factor A	82	33	49	3.7
AGRN	Homo sapiens agrin	503	143	360	3.5
COL3A1	Homo sapiens collagen, type III, alpha 1	145	90	55	3.5
MMP1	Homo sapiens matrix metallopeptidase 1 (interstitial collagenase)	70	29	41	3.3
EGFR	Homo sapiens epidermal growth factor receptor (erythroblasticleukemia viral (v-erb-b) oncogene homolog, avian)	49	79	312	10.4
AFP	Homo sapiens alpha-fetoprotein	391	40	165	9.1

**Figure 2:**
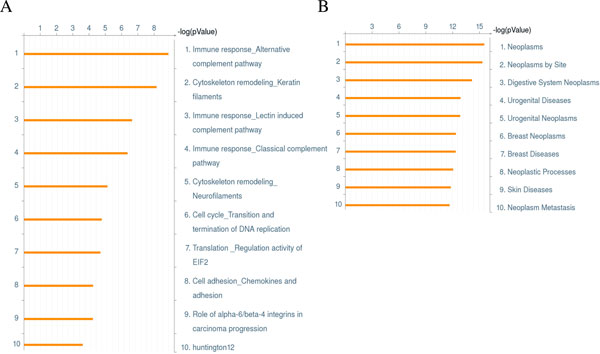
**GeneGo pathway (A) and disease (B) analyses indicated cancer-related genes were enriched**. A. Eight out of 10 pathways enriched are related to cancer. Among these, Pathways 1, 3, and 4 are also related to immune responses. Pathway 2 and 5 are involved with cytoskeleton remodeling. Pathway 6 is transition and termination of DNA replication. Pathway 8 and 9 are adhesion related. B. Out of top 10 most enriched GeneGO disease categories, 7 are cancer related (1-3, 5, 6, 8, and 10). The more significant it is the longer the orange bar. A bar of 15 in length corresponds to a p-value of 1e-15.

To show that this list of 723 genes was enriched for cancer and thus obtains credibility for our methodology, we looked for cancer related pathways associated with them in GeneGO [55] pathways, which covered 650 signaling and metabolic networks (Figure 2A). Among the 10 most significantly matched pathways, several are cancer related - Pathways number 1, 3, and 4 involve immune response; number 2 and 5 involve cytoskeleton remodeling; number 6 is transition and termination of DNA replication; and number 8 and number 9 are adhesion related. In addition, the result of GeneGo disease enrichment analysis (Figure 2B) indicates our set of genes as neoplasm enriched: seven out of the 10 most associated diseases are related to cancer. The disease ranks the highest is neoplasms, followed by neoplasm by site, and digestive systems neoplasm. This list reveals that our 723 DEGs covers general neoplasm related functions, and not specific to any particular neoplasm, as digestive, urogenital and breast are all covered.

To narrow down this list of biomarkers, we crossed examined the expression profiles of the candidates with the differentially expressed genes in 6 microarray experiments, i.e. two each of ovary and uterus, and one each of pancreas and colon (Table 3). These tissue types were selected based on the following reasons. We noticed that many of our candidate genes had the most expression in ovary tissue (after normalization). The other concern was the number of ESTs. Since our candidate genes were derived from EST sampling of various tissue types, they were influenced more heavily by tissue types with more EST representation due to deeper sampling from them. Therefore, the rest of the tissue types were selected based on their representativeness. Of the 723 DEGs, 235 candidates were also found to be differentially expressed genes in our microarray analysis.

**Table 3:** Five microarray projects cross referenced with our set of 723 DEGs

GEO	Tissue type	Test sample size (n vs. c)	Sig genes DN	Reference
GSE18520	Ovary	10 vs. 53	79	[66]
GSE14407	Ovary	12 vs. 12	109	[67]
GSE764	Uterus	4 vs. 7 benign	0	Unpublished
GSE764	Uterus	4 vs. 8 malignant	2	Unpublished
GSE15471	Pancreas	39 vs. 39	120	[68]
GSE23878	Colon	24 vs. 35	74	Unpublished

n: normal, c: cancer

GSE764 has two entries since we compared pair-wise between normal vs. benign and normal vs. malignant.

Since membrane and secretory proteins could be potential therapeutic target or serum biomarkers, the subcellular location of the 235 DEGs were examined against the secretory and membrane protein lists consolidated from public databases. Among these, 96 DEGs were putative membrane or secretory proteins - 57 had only secretory annotation, 27 had only membrane annotation and 12 had both.

### Literature search and STRING analysis of the 96 DEGs

**Figure 3:**
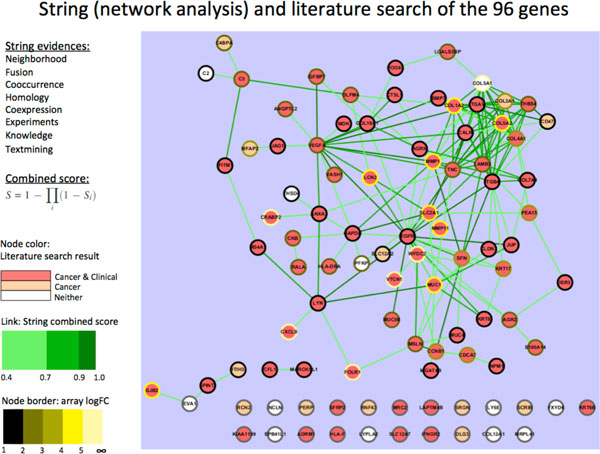
**STRING analysis and literature reviews indicated most candidate genes are highly connected and have published cancer associations**. STRING analysis is based on several evidences indicating the strength of relationships between pairs of proteins. The STRING output was imported into Cytoscape to depict the network. Darker link denotes stronger relationships. Node colors red, apricot, and white denotes literature search with clinical cancer reports, cancer report but not from clinical samples, and no literature search support found, respectively. Lighter border denotes greater logFC of cancer over normal expression in microarray.

To further examine whether the 96 membrane/secretory DEGs identified in our EST database mining had enriched cancer-related genes, we searched the literatures for known associations with cancers. In additions, they were also analyzed with STRING for interactions, which are based on experimental evidence or prediction, such as conserved genomic neighborhood, gene fusion, co-occurrence across genomes, pathways, protein complex, co-regulation, or other literature sources such as co-mentioning. The network of the STRING interactions of the 96 DEGs together with the literature search results were plotted based on the combined STRING score with Cytoscape [56] (Figure 3). Approximately 68 proteins formed a big cluster of interacting proteins and a large proportion of the DEGs (88%) had published cancer association with clinical or non-clinical experimental supports. This demonstrates the value of our integration strategy since we had an ample of literature supports.

**Figure 4:**
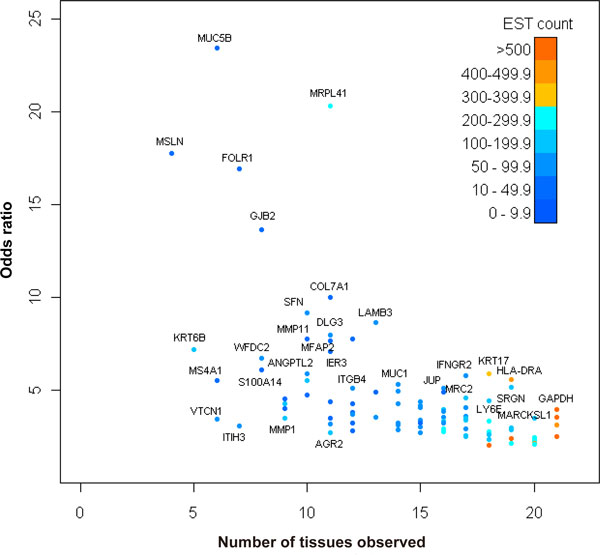
**Distribution of the 96 membrane and secretory DEGs across tissue types**. The 96 DEGs were selected after cross examined with microarray data and membrane and secretory protein lists. A gene is marked as observed in a tissue when at least one EST is from that tissue type. The CMH odds ratios are given to give a perspective of their tendencies of differential expressions. The total EST counts are shown in colors to give a sense of confidence.

The 96 DEGs were selected out of their general cancer propensity without necessarily referring to any particular tissue type. However, we can still assess the general tissue distributions shown in Figure 4. A gene has a tissue representation if any EST from a clone library of the tissue type is matched to it. We can see that some genes are observed across many tissue types. A gene could be observed across a variety of tissue types if it is pan-tissue, and its expression measure is relatively abundant. Separately, Woolf's test for heterogeneity can also give hints to whether a gene is pan-cancer. Those that were found as significant in this test were considered having unequal representation in different genes; although whether they are pan-cancer require further evaluation.

### Three candidates had higher expression in several cancer tissues

**Figure 5:**
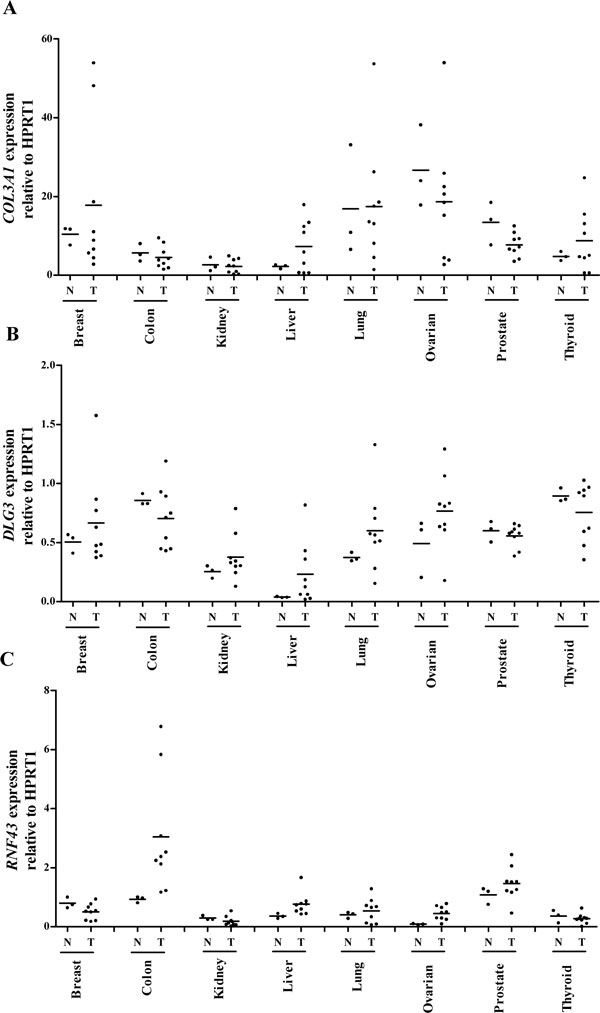
**Relative transcript levels of COL3A1, DLG3, RNF43 in normal and cancer tissues detected by RT-qPCR analysis**. Each dot represents the relative gene expression level normalized against the individual HPRT1 level of each tissue specimen. A total of 3 normal (N) and 9 cancer (T) tissue samples from 8 different tissues or organs were analyzed.

Three cancer differentially expressed secreted protein gene candidates, COL3A1 (Collagen alpha-1(III) chain), DLG3 (Discs large homolog 3), and RNF43 (Ring finger protein 43), which had an odds ratio of 3.55, 7.97, and 4.03, respectively, and with limited or no clinical support were selected for real-time qPCR analysis using the Taqman^® ^Gene Expression Assay kits (Applied Biosystems, Foster City, CA) (Figure 5). With the HPRT1 as the reference, higher expressions of these genes were noticed in at least some of the cancer tissues. Apparently, the average relative expression levels of COL3A1 in breast, liver, thyroid cancer samples were higher than their normal counterparts. The average expression levels of DLG3 in breast, kidney, liver, lung, and ovarian cancers, and RNF43 in colon, liver, lung, ovarian, and prostate cancers were also found to be higher than their normal tissues. The expression of COL3A1 in approximately 5 of the liver cancers, DLG3 in 5 of the liver, 7 lung and 5 ovarian cancers, and RN43 in 7 of the colon, 8 ovarian and 5 prostate cancers seemed to have higher expressions than the normal tissues. In light of the limited sample size, the three candidates appear to have an overall higher expression in cancer tissues.

## Discussion

Reported here is an integrative, meta-analytic approach for the discovery of pan-cancer differentially expressed gene candidates. Our primary enrichment included a set of 723 DEGs with cancer associations supported by GeneGO disease and pathway analysis. Further integrative evaluations with cancer differentially expressed genes suggested by microarray data narrowed the list down to 234 genes, and among these there were 96 DEGs likely belonged to either secretory and membrane protein genes. Further STRING protein network analysis and literature reviewing indicated 71% of the 96 DEGs were highly connected and many of them were associated with cancers in previous publications.

### Simpson's paradox

The meta-analytic nature of our study brought us the opportunities as well as challenges to study the digital signatures of various transcriptomes in a new perspective. Comparing to experimental methods that focus on a single tissue type or limited tissue types, our approach allows us to find genes inclined to express in cancer in a pan-tissue manner. An important challenge of our approach is to avoid Simpson's paradox which can occur in a meta-analysis study [32]. Simpson's paradox is where the association between two variables may show a correlation that is reversed in direction from what is observed from stratified subgroups. A contrived example is shown in Table 1 in which gene A appears to have a higher cancer expression when pooled, but it is in fact not so under individual stratified sub-tables. This may be somewhat of an extreme case, where directionality of the ratios actually differs between the sub-tables and the pooled table. However, the tissue confounder still introduces bias, large or small, that may throw our judgment off. In this study, we used CMH to analyze the data based on stratified sub-tables to avoid running into this paradox. One could also analyze only one tissue at a time for differential expression, but this means one has a smaller dataset to work with. CMH could avoid this problem since it uses EST counts from all tissues instead of analyzing just the normal and cancer propensity under each individual tissue type.

In our actual data, the odds ratio of the pooled table is also different from that of the stratified table. For example, the gene PTRF, a polymerase I and transcript release factor, has a pooled odds ratio of 0.40 and a CMH odds ratio of 0.16 calculated from stratified sub-tables. In this particular case, both odds ratios indicated an inclination toward a higher normal expression and are both statistically significant although at different degree (the pooled has a p-value of 5.269e-15 under a 2x2 χ^2 ^test [57] versus CMH's 7.16E-77). For the gene VCAN (versican), the pooled odds ratio is 1.86 and χ^2 ^test yields a significant p-value of 1.83e-4. However, CMH gives an insignificant result for this gene with p-value of 0.25. As an extreme case, GBP6 (guanylate binding protein family, member 6) has a pooled odds ratio of 6.69 and χ^2 ^test gives a p-value smaller than 2.2e-16 (approaching 0), whereas with CMH the odds ratio is 0.73, actually indicating a higher normal counts, although CMH p-value of 0.15 is insignificant. This indicates Simpson's paradox in action. Careful inspection showed that all cancer counts and most normal counts of GBP6 were contributed by the tongue tissue source. Out of a total of 50 cancer counts and 21 EST normal counts, tongue accounts for cancer and normal counts of 50 and 17, respectively. For this gene, the tongue cancer count 50 is not influential under a total of 29,479 cancer counts and 7,486 counts for the tongue. Thus pooling loses information in this respect and gives a false impression that its cancer expression is much higher when summing all cancer counts from all tissues. Stratifying by tissue type guards against this bias.

### Heterogeneity of odds ratios

In the strictest application, the use of Cochran-Mantel-Haenszel method requires the odds ratios of the sub-tables be homogeneous. In our context, it means the ratio of gene expressions between cancer and normal tissues are probably the same among all tissue types under study and any observed variability is most likely due to sampling bias. Also, the calculated odds ratio would be the estimated common odds ratio across the tissue strata. In our case, however, not all genes had similar ratios under each tissue (based on Woolf's test for homogeneity available in Additional file 2 under the "Woolf" column label), and this was of course expected. In spite of this, we were interested in the overall expression patterns of the genes in cancer conditions. We were not interested in an estimate of common odds ratio across the strata, which often does not exist. We were interested in hypothesis testing - to give us leads to the genes that had higher cancer expression in general. In this regard, the test could be applied [58,59]. The CMH odds ratio is a weighted average of the odds ratio in each tissue classification and can give us a summary measure [60], which we used to prioritize and followed up with subsequent biological analyses. In other word, an odds ratio in our data was merely a value that "average up" across all tissue types. From these ratios we were able to reveal the preferential cancer expressions, since the list covered a number of important known biomarkers, and enrichment of cancer-related genes were supported by knowledge-based GeneGO analyses and previous publications.

### Lower bound of confidence interval

Another distinctive tactic we used is the selection of DEGs among the statistically significant genes (p-value < 0.05) base on lower bounds of the confidence interval of the odds ratio estimates. The popular approach to search for DEGs is to select genes base on p-value first, and then select the subset base on parameter estimators such as odds ratio or fold change values. The p-value criterion selects the statistically significant ones (those not likely to be the result of random fluctuation). The subsequent criterion is based on prior domain knowledge. However, among those with statistically significant p-values and similar parameter estimators, the ranges of the estimations can vary widely. Using our dataset as an example, the two genes TUBA1B and FAM60A both have odd values of 2.38 (Additional file 2). However, for TUBA1B, it is within the 95% confidence that its true odds ratio is between 2.26 and 2.50. Yet for FAM60A it is between 1.59 and 3.54. Based on our background knowledge and for future application, if we must select genes having odds ratios greater than 2.0, then using odds ratio as cutoff would not serve this purpose since it is quite possible that the real odds ratio (i.e., of the population) is below 2.0. Choosing genes based on their confidence intervals would be more precise, but this has not been much appreciated.

### Multi-cancer biomarkers

The multi-cancer approach compares genes that are overall differentially expressed among multiple cancer types comparing to their respective normal tissue types. Although many biomarker studies focus on gene differentially expressed in a particular tissue type, Wu *et al*. found 8 proteins in the conditioned media of 23 cell lines showing negative or weak tissue staining in the Human protein atlas, suggesting them to be potential pan-cancer markers [61]. Sahin *et al*., found that claudin-18 splice variant 2 had the ectopic activations in pancreatic, esophageal, ovarian, and lung tumors while its expression in normal tissue only occurred in differentiated epithelial cells of the gastric mucosa, confirmed by RT-PCR [62]. These studies suggested that relatively multi-cancer genes or multi-cancer splice variants exist. The three candidates COL3A1 (Collagen alpha-1(III) chain), DLG3 (Discs large homolog 3) (plasma membrane), and RNF43 (Ring finger protein 43) are putative secreted or plasma membrane proteins with the potential of developing serum diagnostic reagents. In reviewing the involvement of these genes with cancers in previous studies, hint for pan-cancer marker was surfaced as the expression of the extracellular matrix protein COL3A1 gene in brain cancer [63] and angiofibroma [64] was elevated. While secreted membrane bound RNF43 protein gene was known to be up-regulated in colorectal cancer [65]. Interestingly, upon the real-time qPCR analysis of three cancer differentially expressed secreted protein gene candidates, COL3A1, DLG3, and RNF43 identified in this study, higher cancer expression levels of these genes in multiple cancer types were verified. This does not only indicate the usefulness of our computational approach and filtering procedure but also encourages us to devote further resources for assessing the clinical usages of these three candidates.

### Pooling of gene expression

Earlier in this discussion, we mentioned that naïve pooling of data may introduce bias and at worst may produce Simpson's paradox. We also mentioned that we have tackled this problem with CMH. Nevertheless, two other occasions of pooling actually took place. We pooled expression from different splicing variants from the same gene to make one gene expression. We also pooled different libraries of the same tissue into one tissue classification. In both of these cases, we may encounter expression bias, since different splicing variants and different tissue libraries (i.e., tissues from different patients) might have differences in expression patterns. This is an unfortunate limitation in this and similar studies, since dbEST data consists of many different sources, and given the relative lack of data after the very stringent criteria we have used in our library selection compare to previous studies (Most importantly the exclusion of ESTs from cell lines, PCR amplification, subtraction, and cDNA normalization protocols). We opted for pooling since we had comparatively limited number of sequences to work with (1,644,960 out of 8,296,089 downloaded - 18.03%). Nonetheless, future digital expression profiling can be made better with the RNA-Seq methodology that offers a greater depth of coverage than ESTs obtained from traditional cDNA sequencing. It gives a much larger sampling size that makes more realistic the differentiation among isoforms and also makes pooling of different libraries of the same tissue less necessary. As for discovery of pan-cancer genes or isoforms when studying multiple tissue types, similar idea as outlined in this study would be just as applicable.

## Conclusions

We have demonstrated that the use of the Cochran-Mantel-Haenszel statistic in the integrative approaches allowed us to identify potential biomarkers or therapeutic targets via exhaustive search of various EST libraries from dbEST. As shown in previous study, splice variant could be useful target of antibody therapy [62]. The method can be easily extended over to searching cancer differential splicing variants had there been enough data. The issues involved in the analysis, such as the Simpson's paradox and the pan-cancer markers, may also be encountered in other multi-class digital analysis. The three targets confirmed by real-time qPCR, COL3A1, DLG3, and RNF43, are worthy of further evaluation for clinical applications.

## Competing interests

The authors declare that they have no competing interests.

## Authors' contributions

TW and LC selected and filtered EST libraries as well as STRING analysis. TW conceived the deployment of the statistical method and implemented and ran the EST analysis pipeline. Microarray slides were selected by LC and analyzed by TW. TW, LC, TC were involved with literature search. LC selects the genes for expression validation and STRING analysis. JW performed the real-time qPCR analysis. YT is involved with the statistical interpretation of the results. WL and WN provided direction and guidance. All authors read and approved the final manuscript.

## Supplementary Material

Additional file 1 **Tissue and library distributions of 1,644,960 ESTs**. This table shows the number of ESTs assigned to each tissue type prior to matching to reference sequences. Click here for file

Additional file 2 **EST pipeline raw data**. This is the raw EST count from the EST pipeline imported into Excel. The columns are the condition type, tissue, and condition-tissue type stratifications. The rows represent the EST counts that are assigned to genes. Click here for file
