# Supplementary material for: Meta-analytical biomarker search of EST expression data reveals three differentially expressed candidates
Source: BMC Genomics. 2012 Dec 7;13(Suppl 7):S12. doi: 10.1186/1471-2164-13-S7-S12 (PMC3521215; doi:10.1186/1471-2164-13-S7-S12)
Supplement: Additional file 1 — Tissue and library distributions of 1,644,960 ESTs. This table shows the number of ESTs assigned to each tissue type prior to matching to reference sequences. [file 1471-2164-13-S7-S12-S1.docx]

## Additional file 1 –Tissue and library distributions of 1,644,960 ESTs

This table shows the number of ESTs assigned to each tissue type prior to matching to reference sequences.

| Tissue | EST count | Lib count |  | Tissue | EST count | Lib count |
| --- | --- | --- | --- | --- | --- | --- |
| Brain | 435,782 | 52 |  | Blood | 20,309 | 7 |
| Uterus | 105,233 | 14 |  | Bone | 18,712 | 6 |
| Testis | 97,202 | 11 |  | Artery | 14,162 | 5 |
| Placenta | 71,292 | 16 |  | Bone marrow | 14,089 | 8 |
| Pancreas | 65,692 | 10 |  | Pituitary | 13,242 | 6 |
| Muscle | 63,899 | 9 |  | Cartilage | 12,844 | 4 |
| Liver | 57,863 | 8 |  | Bladder | 11,477 | 2 |
| Kidney | 56,308 | 12 |  | Adipose | 10,802 | 6 |
| Eye | 48,149 | 19 |  | Esophagus | 9,875 | 2 |
| Spleen | 44,857 | 5 |  | Vein | 6,325 | 2 |
| Lung | 43,893 | 15 |  | Thyroid | 6,186 | 5 |
| Skin | 43,381 | 15 |  | Rectum | 5,644 | 2 |
| Ovary | 40,716 | 18 |  | Germ cell | 5,024 | 5 |
| Tongue | 36,965 | 6 |  | Cervix | 4,864 | 2 |
| Colon | 34,925 | 20 |  | Pineal gland | 4,514 | 2 |
| Nerve | 29,338 | 6 |  | T-cell | 2,718 | 3 |
| Lymphoid organ | 28,661 | 11 |  | Salivary | 2,281 | 1 |
| Small intestine | 28,198 | 5 |  | Gallbladder | 2,016 | 2 |
| Prostate | 28,192 | 20 |  | Head and neck | 1,336 | 3 |
| Pharynx | 24,208 | 4 |  | Leukemia | 1,114 | 1 |
| Adrenal gland | 23,716 | 11 |  | Larynx | 733 | 1 |
| Stomach | 23,263 | 4 |  | Thymus | 587 | 1 |
| Hart | 22,179 | 6 |  | Mesenchymal | 468 | 1 |
| Beast | 21,296 | 14 |  | Spinal cord | 430 | 1 |
